# Post-traumatic stress impact on health outcomes in Gulf War Illness

**DOI:** 10.1186/s40359-021-00561-2

**Published:** 2021-04-20

**Authors:** Mary Jeffrey, Fanny Collado, Jeffrey Kibler, Christian DeLucia, Steven Messer, Nancy Klimas, Travis J. A. Craddock

**Affiliations:** 1 Institute for Neuro-Immune Medicine, Nova Southeastern University, Fort Lauderdale, FL USA; 2 Department of Psychology and Neuroscience, Nova Southeastern University, Fort Lauderdale, FL USA; 3 Miami Veterans Affairs Medical Center, Miami, USA; 4 Department of Clinical and School Psychology, Nova Southeastern University, Fort Lauderdale, FL USA; 5 Department of Clinical Immunology, Nova Southeastern University, Fort Lauderdale, FL USA; 6 Department of Computer Science, Nova Southeastern University, Fort Lauderdale, FL USA

**Keywords:** Gulf War Illness, Post-traumatic stress disorder, Co-morbid conditions, Hierarchical regression, Sub-typing

## Abstract

**Background:**

Gulf War Illness (GWI) is a chronic, multi-symptomatic disorder affecting an estimated 25–32% of the returning military veterans of the 1990–1991 Persian Gulf War. GWI presents with a wide range of symptoms including fatigue, muscle pain, cognitive problems, insomnia, rashes and gastrointestinal issues and continues to be a poorly understood illness. This heterogeneity of GWI symptom presentation complicates diagnosis as well as the identification of effective treatments. Defining subgroups of the illness may help alleviate these complications. Our aim is to determine if GWI can be divided into distinct subgroups based on PTSD symptom presentation.

**Methods:**

Veterans diagnosed with GWI (*n* = 47) and healthy sedentary veteran controls (*n* = 52) were recruited through the Miami Affairs (VA) Medical Health Center. Symptoms were assessed via the RAND short form health survey (36), the multidimensional fatigue inventory, and the Davidson trauma scale. Hierarchal regression modeling was performed on measures of health and fatigue with PTSD symptoms as a covariate. This was followed by univariate analyses conducted with two separate GWI groups based on a cut-point of 70 for their total Davidson Trauma Scale value and performing heteroscedastic t-tests across all measures.

**Results:**

Overall analyses returned two symptom-based subgroups differing significantly across all health and trauma symptoms. These subgroups supported PTSD symptomatology as a means to subgroup veterans. Hierarchical models showed that GWI and levels of PTSD symptoms both impact measures of physical, social, and emotional consequences of poor health (*ΔR*^2 ^= 0.055–0.316). However, GWI appeared to contribute more to fatigue measures. Cut-point analysis retained worse health outcomes across all measures for GWI with PTSD symptoms compared to those without PTSD symptoms, and healthy controls. Significant differences were observed in mental and emotional measures.

**Conclusions:**

Therefore, this research supports the idea that comorbid GWI and PTSD symptoms lead to worse health outcomes, while demonstrating how GWI and PTSD symptoms may uniquely contribute to clinical presentation.

**Supplementary Information:**

The online version contains supplementary material available at 10.1186/s40359-021-00561-2.

## Background

Gulf War Illness (GWI) is a prevalent health condition impacting 25–32% of the 700,000 deployed US military personnel to the 1990–1991 Gulf War [27]. GWI typically presents with some combination of fatigue, pain, headache, difficulty concentrating, memory loss, sleep disturbance, respiratory issues, gastrointestinal problems, and skin rash [17]. GWI is multifaceted, as it impacts multiple systems of the body (i.e., autonomic nervous system, endocrine system, and immune system), leading to various presentations of those diagnosed with GWI [27]. As such, GWI etiology remains elusive, halting the efficacy of diagnosis and treatment.

Veterans with GWI symptoms experience continuous challenges in dealing with their health and day to day functioning [15, 34]. Twenty years following the Gulf War, GWVs continue to report poor health outcomes, with higher prevalence rates of both physical and mental health conditions [9]. Additionally, GWVs are more likely to endorse poor health status and reported greater disability. Kang et al. [20] found that GWVs reported poor health status (i.e., only 35% of GWVs endorsed “very good” health) and suffered economically from their illness (i.e., using more sick days, increased visits to the physician) in comparison to veterans who were not deployed. In a structured equation model of GWI, Iannacchione et al. [19] found that impaired cognition, confusion-ataxia, and central pain were significant contributors to a decrease in functionality observed between GWVs and non-deployed veterans. Therefore, GWI remains a vital research subject in efforts to provide relief to GWVs suffering from GWI and to help improve functionality.

GWI presentation can be impacted by co-morbid conditions, particularly post-traumatic stress disorder (PTSD). Studies indicate that approximately 29–39% of veterans develop post-traumatic stress disorder (PTSD; PTSD; [1, 21, 32, 36]) a psychological condition characterized by exposure to a psychologically distressing event (e.g., combat) that causes symptoms of re-experiencing (e.g., flashbacks, nightmares, frightening thoughts), avoidance (e.g., emotional numbness, avoidance of events, places, or objects,), hyperarousal (e.g., feeling tense, being easily startled, and difficulty sleeping) and cognitive/mood symptoms (e.g., difficulty remembering the traumatic event, anhedonia, negative beliefs about oneself and the world [2]). Trauma exposure is a significant concern, as it leads to poor health outcomes in veterans. When investigating PTSD in GWVs, Barrett et al. [3] found that endorsements of fair to poor health statuses were common, as were lower quality of life due to health-related difficulties. Gade and Wenger [15] found that GWVs exposed to dead, dying, or injured people exhibited a higher need for mental health services, even after controlling for demographic characteristics, social economic status, and insurance coverage. Engel et al. [11] investigated how PTSD impacted physical symptom reporting amongst GWVs, and found that GWVs diagnosed with PTSD reported more physical health symptoms over veterans with other psychiatric disorders and healthy veterans. Wachen et al. [34] found significantly increased physical symptoms in veterans with posttraumatic stress symptomatology as measured before and after the war. Posttraumatic stress symptomatology was identified as a mediator between warzone exposure and physical health, impacting all physical health domains tested (i.e., cardiovascular, dermatological, gastrointestinal, genitourinary, musculoskeletal, neurological, and pulmonary). Therefore, PTSD is a significant concern when considering GWI as it is also contributing to poor health presentation.

Given the combative nature of warfare, Gulf War Veterans (GWVs) have a higher risk of developing PTSD symptoms along with contracting GWI. Although PTSD has a lower prevalence rate compared to other combat veteran populations, research indicates that exposure to trauma in the Gulf War consequently led to higher utilization of mental health services and poorer health outcomes [15, 34]. However, research remains unclear how differential levels of PTSD symptoms specifically contributes to GWI presentation [13, 18]. This study was designed to clarify the patterns between the burden of PTSD symptoms and GWI through investigating health outcome measures between veterans grouped by diagnostic criteria (i.e., with or without GWI) and level of PTSD symptoms.

As the heterogeneity of GWI symptom presentation complicates diagnosis as well as the identification of effective treatments, defining subgroups of the illness may help alleviate these complications. Here we aim to determine if GWI can be divided into distinct subgroups based on PTSD symptom presentation by performing a secondary analysis on pre-existing data. First, hierarchal regression modeling was performed on measures of health and fatigue with PTSD symptoms as a covariate to determine how PTSD symptoms affect overall GWI symptom presentation. Univariate analysis was then conducted on two separate GWI groups based on a cut-point of 70 for their total Davidson Trauma Scale value followed by heteroscedastic t-tests across all measures.

## Methods

### Ethics statement

All participants signed an informed consent approved by the Institutional Review Board of the Miami Veterans Affairs Medical Center. Ethics review and approval for data analysis was also obtained by the IRB of Nova Southeastern University**.**

### Participants/procedure

Participants were recruited via the Miami Veterans Affairs (VA) Medical Health Center in two cohorts funded under a Veterans Affairs Merit award (GWI: *n* = 23, healthy controls (HC): *n* = 27) and a Department of Defense Gulf War Illness Research Program award (W81XWH-09-2-0071) (GWI: *n* = 24, HC: *n* = 25); both which compared male veterans with GWI to HC. Therefore, the full sample for the multivariate and univariate analyses included *n* = 47 male veterans with GWI in addition to *n* = 52 healthy controls. Demographics are presented in Table 1. All participants were male with an average age of 43.47 years with an average BMI of 29.84. The sample consisted of 26.3% White, 23.2% Black, 47.5% Hispanic, 2.0% Asian, and 1% Other males. Inclusion criteria for GWI participants was derived from Fukuda et al. [14] and consisted in identifying veterans deployed to the theater of operations between August 8, 1990 and July 31, 1991, with one or more symptoms present for 6 months from at least 2 of the following: fatigue, mood and cognitive complaints; and musculoskeletal complaints. Participants were in good health prior to 1990 and had no current exclusionary diagnoses defined by Reeves et al. [26]. This includes exclusion of major dementias of any type and alcoholism or drug abuse, medical conditions including organ failure, rheumatologic disorders, and use of medications that impact immune function, such as steroids or immunosuppressives. Collins et al. [6] supports the use of the Fukuda definition in GWI. Control participants consisted of Gulf War era veterans self-defined as healthy with no exclusionary diagnoses, and sedentary (no regular exercise program, sedentary employment).

**Table 1:** Demographics of cohort

Group	Total	GWI	GWI +	GWI −	HC	p_2_	p_3_
N	99	47	32	15	52		
Mean age	43.47 ± 6.30	43.40 ± 5.44	44.38 ± 5.00	41.33 ± 5.95	43.54 ± 7.04	0.851	0.304
Mean BMI	29.84 ± 4.62	30.70 ± 4.46	30.28 ± 4.12	31.60 ± 5.12	29.06 ± 4.68	0.078	0.139
*Race*						0.170	0.313
White	26.30%	27.70%	21.90%	40.00%	25.00%		
Black	23.20%	29.80%	34.40%	20.00%	17.30%		
Hispanic	47.50%	38.30%	40.60%	33.30%	55.80%		
Asian	2.00%	4.30%	3.10%	6.70%	0.00%		
Other	1.00%	0.00%	0.00%	0.00%	1.90%		
*Marital status*						0.232	0.640
Married	62.63%	70.21%	71.88%	66.67%	55.77%		
Widowed	1.01%	0.00%	0.00%	0.00%	1.92%		
Divorced	4.04%	4.26%	3.13%	6.67%	3.85%		
Separated	12.12%	14.89%	12.50%	20.00%	9.62%		
Never married	13.13%	8.51%	9.38%	6.67%	17.31%		
Not answered	7.07%	2.13%	3.13%	0.00%	11.54%		
Employed	77.78%	76.60%	68.75%	93.33%	78.85%	0.788	0.162
Avg. school years	15.41 ± 2.49	15.34 ± 2.28	15.38 ± 2.01	15.27 ± 2.84	15.48 ± 2.71	0.783	0.956

### Measures

All participants received a physical examination and medical history including the GWI symptom checklist as per the case definition. Symptom questionnaires included the Multidimensional Fatigue Inventory (MFI, [29], α = 0.953), a 20-item self-report instrument designed to measure fatigue with 5 resulting composite scores, the RAND Medical Outcomes Study 36-item short-form survey (RAND SF-36, [35], α = 0.954) assessing health-related quality of life with 8 resulting composite scores, and the Davidson Trauma Scale (DTS; [8]), a self-rating measurement of the frequency and severity of PTSD symptoms in three clusters: intrusion, avoidance, and hyperarousal.

### Data analysis

#### Hierarchical multiple regression analysis

The hierarchical regression analyses were conducted through multiple steps (*n* = 47 GWI, and *n* = 52 HC) to measure how GWI status and PTSD symptoms would impact self-reported levels of health issues (RAND SF-36) and fatigue (MFI). Several assumption tests were performed to prevent Type 1 error (Additional file 1). As age and BMI are both known to affect the overall reporting on the RAND SF-36 and MFI scales [16, 38], a one-way ANOVA with age and BMI was conducted between healthy controls and veterans with GWI to test for potential confounding factors. While there was no significant difference between the control and GWI veterans by age *F*(1, 96) = 0.021, *p* = 0.913, or BMI, *F*(1, 96) = 3.170, *p* = 0.078, age and BMI were nevertheless controlled for within the hierarchical regression analyses. BMI and Age were the first variables in the model (Block 1) to control for demographic variables. The next block (Block 2) held the categorical variable of health condition as defined by either a healthy control or a participant with GWI. The last block contained the continuous variable for level of PTSD symptoms as measured by the total DTS score (Block 3). All three blocks were held consistent when applied to all eight subscales of the RAND-36 and the five subscales of the MFI. Assumptions were tested for all measures (i.e., RAND SF-36 and MFI) and analyses of the data were analyzed via SPSS Version 25 (IMB Corporation, 2017). All analyses were interpreted with the alpha level of 0.05. Additionally, effect sizes for GWI and PTSD symptom levels were determined using multiple R^2^ change cutoffs and squared semi-partial correlations (*r*_*sp*_^2^) as determined by [5] with a negligible effect being smaller than 0.02, a small effect being above 0.02, a medium effect being above 0.13, and a large effect being 0.26 or higher. Finally, missing data was minimal (maximum percentile missing = 7.0%) and coded as missing. Additionally, analyses were run in a pairwise fashion.

#### Cut-point analysis

The DTS is a 17-item self-report questionnaire of post-traumatic stress symptoms [7, 8] corresponding to the DSM-IV symptoms of PTSD. A total score, reflecting both frequency and severity ratings for all 17 items and separate ratings for the total frequency and total severity of all 17 items, was used to interpret PTSD probability. The 3 clusters—Intrusive, Avoidance/Numbing, and Hyperarousal—were also scored separately. Following McDonald et al. [22] we applied a simple cut score at 70 for the total DTS score as it has been shown to offer optimal diagnostic accuracy, correctly classifying 90% of cases and provided an accurate estimate of PTSD population prevalence (12–13%). Those GWI subjects with DTS scores 70 and above were considered as probable PTSD positive (GWI +), while those below 70 were considered as probable PTSD negative (GWI −).

Following cut-point group assignment, values were compared between groups using two sample t-tests with unequal variances (i.e. heteroscedastic). Multiple comparisons were corrected using the linear step-up procedure introduced by Benjamini and Hochberg [4]. The effect size of the difference between groups for each measure were also estimated using the corrected Hedges g^*^, where

$${g}^{*}=\left(1-\frac{3}{4\left({n}_{1}+{n}_{2}\right)-9}\right)\frac{{\bar{x}}_{1}-{\bar{x}}_{2}}{{s}^{*}}$$

with $\bar{x}$ being the mean value of the variable for a group, n being the size of the group, and s^*^ the pooled standard deviation for the variable defined as,

$${s}^{*}=\sqrt{\frac{\left({n}_{1}-1\right){s}_{1}^{2}+({n}_{2}-1){s}_{2}^{2}}{{n}_{1}+{n}_{2}-2}}$$

where ${s}^{2}$ is the group variance for the variable. The corrected Hedges g^*^ was chosen as it gives better estimates for small sample size and is corrected to account for bias as an estimator for the population effect size. Effect sizes were interpreted in the following ranges [28]: negligible, lower than 0.01, very small, 0.01–0.20, small 0.20–0.50, medium 0.50–0.80, large 0.80–1.20, very large 1.20–2.00, and huge 2.00 or higher.

## Results

### Demographics

Demographics for the cohort are given in Table 1. Statistical comparisons were made between both GWI and HC groups (p_2_), as well as between the GWI + , GWI −, and HC groups (p_3_) using ANOVA for continuous variables and the χ^2^ test for categorical variables. No statistical differences were found in age, BMI, racial representation, marital status, employment status, or average number of years in school.

### Hierarchical multiple regression

In the first set of hierarchical regression models, the eights scales of the RAND SF-36 were analyzed in separate models. Predictor blocks were held constant across models: Block 1 (Age, BMI), Block 2 (GWI or Healthy Control) and Block 3 (PTSD Symptoms). Hierarchical regression models were constructed with three blocks of predictor variables to assess their effect on each of the 5 scales of the MFI, and the 8 scales of the RAND SF-36. A summary of the increase in the Coefficients of Determination (R^2^) of the symptom measures for each model is presented in Table 2. Full statistics for each model are provided in the Additional file 1.

**Table 2:** Hierarchical multiple regression model increases in coefficient of determination results for age and BMI (Model 1), with GWI Health Status (Model 2), and with DTS Total Score (Model 3)

Measure	Model 1	Model 2	Model 3
	*Δ R*^*2*^	*Δ R*^*2*^	*Δ R*^*2*^
Physical functioning	0.112*	0.250**	0.167**
Physical role^a^	0.071	0.435**	0.085**
General health perceptions	0.095**	0.436**	0.091**
Energy/fatigue (Vitality)	0.122**	0.418**	0.154**
Social functioning	0.125**	0.327**	0.218**
Emotional role^b^	0.059	0.359**	0.248**
Emotional well-being	0.073*	0.425**	0.181**
Pain	0.111**	0.338**	0.164**
Physical fatigue	0.106**	0.418**	0.066**
Mental fatigue	0.081*	0.439**	0.055**
Reduced activity	0.155**	0.354**	0.316**
Reduced motivation	0.062*	0.343**	0.131**
General fatigue	0.111**	0.448**	0.131**

*p < 0.05; **p < 0.01

^a^Physical Role (Role Limitations due to Physical Health)

^b^Emotional Role (Role Limitations due to Emotional Problems)

Hierarchical multiple regression modelling showed that combined BMI and age had a significant medium effect the RAND SF-36 measure of Reduced Activity. The RAND SF-36 Role Limitations to Physical Health, and Role Limitations due to Emotional Problems showed no significant effect due to BMI and age. The remaining RAND SF-36 and MFI scores all had a small significant effect due to BMI and age.

The greatest overall contribution to the RAND SF-36 and MFI measures was found to be the addition of GWI status. All measures were shown to have large significant effects due to GWI health status. Yet, while GWI status was shown to have the greatest effect on symptom reporting PTSD symptoms were also shown to have variable non-negligible effects on all the measures. PTSD symptoms were shown to have significant large effects on the MFI measure of Reduced Activity. PTSD symptoms were also shown to have significant medium effects on the RAND SF-36 measures of Physical Functioning, Energy/Fatigue (Vitality), Social Functioning, Role Limitations due to Emotional Problems, and Emotional Well-Being as well as the MFI measures of Pain, Reduced Motivation and General Fatigue. Significant small effects of PTSD symptoms were also found in the RAND SF-36 Role Limitations due to Physical Health, General Health Perceptions, and the MFI measures of Physical and Mental Fatigue.

### Cut-point analysis

**Fig. 1:**
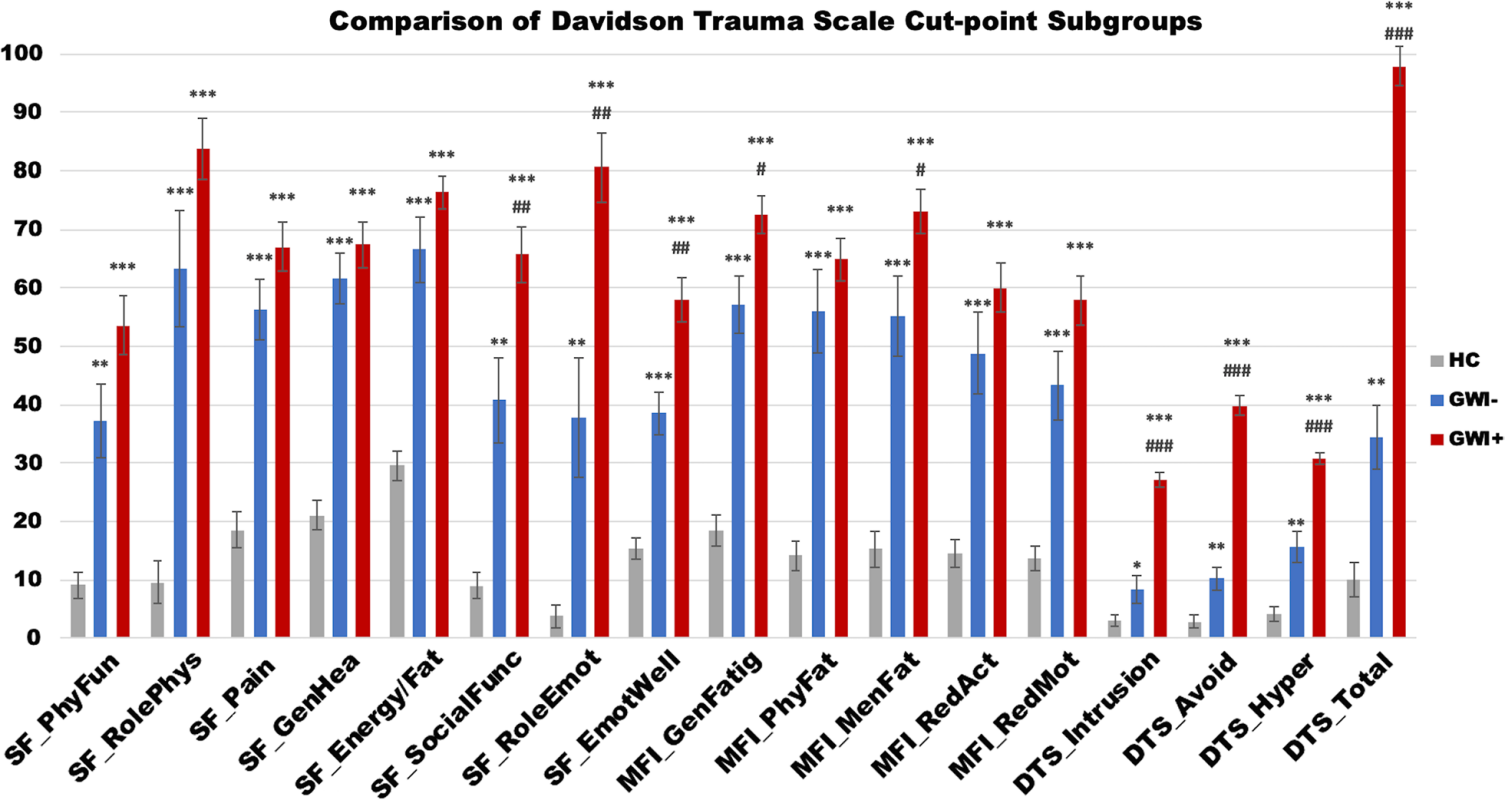
Comparison of symptom scales, and PTSD symptom level scores between Davidson Trauma Scale Cut-point defined groups. *Note* SF-36 scores shown as (100 − score) to invert scale to align with MFI and DTS such that higher values indicate greater disability. SEM error bars. *p < 0.05; **p < 0.01; ***p < 0.001 as compared to HC via heteroscedastic two sample t-test, ^#^*p* < 0.05; ^##^*p* < 0.01; ^###^*p* < 0.001 as compared to GWI − via heteroscedastic two sample t-test

Both GWI subgroups showed significantly higher values in all measures of the SF-36, MFI and DTS scales compared to healthy controls (Fig. 1; Table 3). Compared to GWI − the GWI + group showed a general trend of higher values in all measures with significantly higher values in SF-36 Social Functioning, SF-36 Role Limitations due to Emotional Problems, SF-36 Emotional Wellbeing, MFI General Fatigue, and MFI Mental Fatigue.

**Table 3:** Comparison of Davidson Trauma Scale cut-point derived GWI subgroups

Measure	Mean (SEM)			p^c^			g*		
	HC	GWI −	GWI +	HC /GWI −	HC /GWI +	GWI − /GWI +	HC /GWI −	HC /GWI +	GWI− /GWI +
RAND SF-36*									
Physical functioning	9.2 (2.2)	37.3 (6.3)	53.7 (5.0)	0.001	< 0.001	0.053	7.56	12.05	2.96
Physical role^a^	9.7 (3.7)	63.3 (10.0)	83.9 (5.2)	< 0.001	< 0.001	0.083	8.92	16.70	2.87
Pain	18.6 (3.0)	56.3 (5.2)	67.1 (4.1)	< 0.001	< 0.001	0.116	10.17	13.67	2.37
General health percept	21.1 (2.5)	61.7 (4.4)	67.5 (3.9)	< 0.001	< 0.001	0.326	13.00	14.54	1.42
Energy/fatigue	29.6 (2.6)	66.7 (5.6)	76.4 (2.8)	< 0.001	< 0.001	0.140	10.28	17.29	2.44
Social function	9.1 (2.2)	40.8 (7.3)	65.7 (4.8)	0.001	< 0.001	0.008	7.68	15.86	4.30
Emotional role ^b^	3.8 (1.9)	37.8 (10.2)	80.6 (5.8)	0.005	< 0.001	0.001	6.28	18.72	5.63
Emotional well-being	15.5 (1.8)	38.6 (3.6)	58.0 (3.8)	< 0.001	< 0.001	0.001	9.69	15.10	5.12
*MFI*									
General fatigue	18.5 (2.6)	57.2 (4.9)	72.6 (3.3)	< 0.001	< 0.001	0.016	11.57	18.44	3.87
Physical fatigue	14.1 (2.5)	56.0 (7.1)	65.0 (3.7)	< 0.001	< 0.001	0.287	9.89	16.47	1.77
Mental fatigue	15.3 (3.1)	55.3 (6.8)	73.2 (3.7)	< 0.001	< 0.001	0.031	9.08	16.83	3.59
Reduced activity	14.6 (2.4)	48.8 (7.0)	60.1 (4.3)	< 0.001	< 0.001	0.196	8.29	13.55	2.10
Reduced motivation	13.7 (2.0)	43.3 (5.8)	57.9 (4.3)	< 0.001	< 0.001	0.054	8.64	13.84	2.99
*DTS*									
Intrusion	3.0 (1.0)	8.4 (2.3)	27.2 (1.2)	0.043	< 0.001	< 0.001	3.77	22.73	11.60
Avoidance/numbness	2.9 (1.1)	10.3 (1.9)	39.9 (1.7)	0.003	< 0.001	< 0.001	5.27	26.23	16.50
Hyperarousal	4.1 (1.3)	15.7 (2.6)	30.8 (1.1)	0.001	< 0.001	< 0.001	6.68	21.92	8.71
Total	10.1 (3.0)	34.4 (5.5)	97.9 (3.3)	0.001	< 0.001	< 0.001	6.43	28.08	15.27

^*^SF-36 scores shown as (100 − score) to invert scale to align with MFI and DTS such that higher values indicate greater disability

^a^Physical Role (Role Limitations due to Physical Health)

^b^Emotional Role (Role Limitations due to Emotional Problems)

^c^False discovery rate calculated by the method of Benjamini and Hochberg [4] for all significant *p* values is < 0.06

Substantial effect differences were also observed (Table 3). GWI − and GWI + status showed huge effect differences compared to controls across all measures. Between GWI − and GWI + the presence of higher PTSD symptoms showed effect differences that were again huge for all measures except the SF-36 General Health Perceptions and the MFI Physical Fatigue measures which showed very large effect sizes between the high and low PTSD symptom GWI subgroups.

## Discussion

As PTSD has led to worse health outcomes and has potential impact on the immune system [14, 29–33, 37], there is a possibility that trauma can exacerbate the GWI symptomatology, producing more pronounced health-related consequence. Some research has focused on treating PTSD as a covariate of GWI to be controlled for statistically therefore, there is a lack of research on health outcomes in veterans’ groups specifically by GWI and PTSD. In addition, research has been inconsistent in defining the severity of PTSD and its relationship to health outcomes in GWVs. This study was designed to investigate GWVs diagnosed with GWI presenting with or without comorbid trauma symptomatology on health outcome measures when compared to veteran controls.

It was hypothesized that co-morbid GWI and high PTSD symptom levels will lead to the highest endorsement of problematic mental and physical health outcomes over GWI and posttraumatic stress as separate conditions. The hierarchical model of co-morbid GWI and PTSD symptoms was significant for all domains of reported health outcomes measures. Notably, the GWI diagnosis was particularly influential in impacting health outcomes including physical functioning, role limitations due to physical health, general health, energy/fatigue levels, social functioning, role limitations due to emotional problems, emotional well-being, and pain. In fact, virtually all of these domains revealed that GWI had the largest effect on these score outcomes, with the exception of physical functioning which maintained a medium effect. PTSD symptoms also influenced reported health outcome above and beyond the influence of GWI in all domains but most especially in worse physical functioning, energy/fatigue (vitality), social functioning, emotional well-being, and pain as PTSD symptoms maintained a medium effect. Although still notable, PTSD symptoms had a small effect on role limitations due to physical health and general health. On a measure of fatigue, a model of GWI and PTSD symptom was significant for all measured domains. Additionally, GWI accounted for significantly large amount of variance in physical fatigue, mental fatigue, reduced activity, reduced motivation, and general fatigue. PTSD symptom also showed a medium impact on scores including reduced motivation, mental fatigue, and general fatigue. A small impact was shown in measures of physical fatigue and reduced activity, which may have a more organic basis. Overall, these results show that the presence of PTSD symptoms worsen the symptoms associated with GWI, suggesting a spectrum effect such that the greater the PTSD symptomatology the greater the effect on GWI symptom presentation.

Per Nicolson et al. [23], veterans suffering from GWI are often misdiagnosed with PTSD, assuming that their symptoms are a somatic reaction to trauma with no underlying organic basis. The differentiation between GWI and PTSD may also complicate diagnosis for physicians, as stress also has an impact on biological processes. Specifically, PTSD prompts a prolonged defense mechanism that increases cardiovascular output as a survival response, which simultaneously suppresses the immune system [25]. Therefore, the physical symptoms from suppressed immunological functioning can lead to many of the symptoms reported by GWI veterans [12]. However, the current research presents a strong argument for GWI as a stand-alone diagnosis. The hierarchical regression analysis showed that GWI status was the primary driver of symptom presentation with PTSD symptoms exacerbating this presentation. Furthermore, results from the cut-point analysis show that GWI with a probable negative PTSD diagnosis for a military population still present with a huge symptom burden compared to controls. The analysis with the cut-point in the DTS total score demarcates the GWI groups primarily in the mental and emotional symptom measures, but all symptom measures being exacerbated further in the presence of past traumatic stress as evidenced by the very large effect size differences between measures. This makes sense of GWVs who deny exposure to trauma continuing to report GWI symptomatology. Recent literature has also established a causal link between toxin exposure and GWI onset, using retrospective analyses with GWVs and animal model studies [27]. Therefore, it is unlikely that psychological distress alone is the sole underlying cause of GWI symptoms.

Overall the findings presented here are consistent with research by Gade and Wegner [15] which reported a decline in functioning in GWI with poorer outcomes associated in veterans with GWI and PTSD symptom. Additionally, poorer health outcomes in GWI and PTSD match results as reported by Barrett et al. [3] who found that GWI veterans had lower quality of life due to health-related difficulties. These results as well as higher levels of fatigue as seen in previous research may also be contributing to results stipulated in Kang et al. [20] where GWVs with poor health statuses were more likely to suffer economically with reduced work and higher resources allocated to health services. Regarding specific findings, reductions in mental fatigue found in GWI and PTSD symptom could be linked to findings by Iannacchione et al. [19] who found that impaired cognition was a main factor in decreased functionality in GWVs. Additionally, these findings that show higher variance accounted for by GWI and PTSD symptom in physical functioning and physical role functioning is consistent with Engel et al. [11] as GWVs diagnosed with PTSD were more likely to report more physical health symptoms.

The findings presented here are also consistent with Engdahl et al. [10] which showed that for all symptom domains, severity was highest for GWI with a mental health condition (PTSD (64%), mood disorders (57%), non-PTSD anxiety disorders (11%), other diagnosis (17%)), lower for the GWI group, and lowest for the control group. However, in addition to an increase in symptom burden Engdahl et al. [10] also identified group differences in brain function between health, GWI, and GWI with a mental health condition, which is suggested to be mediated by changes in expression of intercellular cell adhesion molecule 5 (ICAM-5) which modulates synapse formation, immune function and inflammation. In addition to this Niles et al. [24] found that GWI veterans with higher levels of PTSD symptom exposure and current PTSD show autonomic changes in elevated heart rate and skin conductance compared to those without PTSD [24]. As immune dysregulation can serve as a mediator between these autonomic stress reactions, brain function, and health problems among individuals who have experienced traumatic stress, this suggests that the underlying immune profiles of participants with GWI alone may differ from GWI participants co-morbid with PTSD. Future research investigating biological data particularly those sensitive to toxin exposure and immunological functioning would greatly aid the clinical picture of GWI and PTSD symptom. Therefore, it is our hope that this research prompts further investigation and also helps inform clinicians about additional patterns they may encounter in the GWV population.

Several limitations are notable within this study. Due to the secondary nature of the analysis, control of extraneous variance was restricted to post-hoc analysis. Additionally, these samples were relatively small particularly for multivariate analyses. Finally, results did show that the difference amongst male GWI and HC groups in BMI approached significance, however due to the known effect of BMI on the SF-36 and MFI measures this was controlled for in the analyses. Despite these limitations these findings highlight the importance of recognizing how GWI and PTSD symptom may interact with a veteran’s health independently and also how it might present clinically when these conditions are co-morbid.

## Conclusions

We hypothesized that GWI co-morbid with high traumatic stress levels would lead to higher endorsement of problematic mental and physical health outcomes over GWI alone as a separate condition. Specifically, this study found that GWI itself has a profound influence on reported health and fatigue, with related physical, social, pain, and emotional factors. In the presence of past PTSD symptom these symptoms are exacerbated. Overall, the results presented here support the hypothesis that GWI and PTSD symptom both contribute uniquely to the reported functioning impacted by psychological and physical symptoms as well as a subjective experience of fatigue independent of age and BMI status.

## Supplementary Information

**Additional file 1.** Assumptions and hierarchical regression analysis statistics.

## Data Availability

The datasets used and/or analyzed during the current study are available from the corresponding author on reasonable request, pending proper approvals.

## Abbreviations

BMI: Body Mass Index
DTS: Davidson Trauma Scale
GWI: Gulf War Illness
GWVs: Gulf War Veterans
GWI +: High PTSD symptom GWI cut-point subgroup
GWI −: Low PTSD symptom GWI cut-point subgroup
HC: Healthy controls
MFI: Multidimensional Fatigue Inventory
PTSD: Post-traumatic stress disorder
RAC-GWVI: Research Advisory Committee on Gulf War Veteran’s Illness
RAND SF-36: RAND Medical Outcomes Study 36-item short-form survey
UML: Unsupervised machine learning
VA: Veteran affairs
