# Supplementary material for: Post-traumatic stress impact on health outcomes in Gulf War Illness
Source: BMC Psychol. 2021 Apr 20;9:57. doi: 10.1186/s40359-021-00561-2 (PMC8056666; doi:10.1186/s40359-021-00561-2)
Supplement: Supplementary file 1 — Additional file 1. Assumptions and hierarchical regression analysis statistics. [file 40359_2021_561_MOESM1_ESM.docx]

**Gulf War Illness and Post-Traumatic Stress: Impact on Health Outcomes**

Mary Jeffrey, Ricardo Castellanos, Jeffrey Kibler, Christian DeLucia, Steven Messer, Nancy Klimas, Travis J. A. Craddock^*^

*Corresponding author: Travis J.A. Craddock; tcraddock@nova.edu

**Supplementary Material**

**Assumptions**

Independence of observations using the Durbin-Watson statistic (i.e., acceptance range from 0 to 4, with numbers near 2 reflective of no correlation between residuals). The following measures from the RAND SF-36 (see Table S1) and MFI (see Table S2) had independence of observations, as assessed by a Durbin-Watson statistic.

The assumption of linearity between trauma and all dependent variables in a hierarchical regression was assessed collectively with a scatterplot of studentized residuals against unstandardized predicted values. These scatterplots were also used to assess homoscedasticity. All scatterplots met the assumption of linearity (collective) and homoscedasticity; however, RAND SF-36 Role limitations due to physical health, role limitations due to emotional problems, and emotional well-being showed possible mild violations. The assumption of linearity for trauma itself was assessed with partial regression plots between DTS scores and all dependent variables. Visual inspection of all partial regression plots indicates this assumption was met.

Multicollinearity was assessed with correlations (i.e., no correlations between independent variables greater than 0.7) and tolerance values (i.e., no values less than 0.1). All tolerance levels were above 0.1 for each variable, which does not indicate a multicollinearity violation. Outliers were identified with case wise diagnostics. However, the outliers did not show dangerous leverage (all under 0.50) or have significant influence (i.e., no value above 1). Therefore, the outlier remained in the analyses. Finally, normality of residuals was assessed via visual inspection of histograms. Normality was relatively intact. Additionally, regression is robust against minor violations; therefore, no transformations were applied to the data.

Table S1

*Durbin-Watson Statistics in RAND SF-36*

| RAND SF-36 Measures |  |
| --- | --- |
| Physical Functioning | 2.002 |
| Role Limitations Physical Health | 2.102 |
| Energy/Fatigue | 2.091 |
| Social Functioning | 2.002 |
| Role Limitations Emotional | 2.057 |
| Emotional Well-Being | 1.824 |
| Pain | 1.954 |
| General Health | 2.020 |

Table S2

*Durbin-Watson Statistics in MFI*

| MFI Measures |  |
| --- | --- |
| Physical Fatigue | 2.258 |
| Mental Fatigue | 2.011 |
| Reduced Activity | 2.056 |
| Reduced Motivation | 2.042 |
| General Fatigue | 2.003 |

**Hierarchical Regression Analysis Statistics**

| **RAND SF-36: Results:** Hierarchical multiple regression models were on available data (*n* = 94). The full model for RAND SF-36 physical functioning was statistically significant, *R^2^* = .530, *F*(4, 89) = 25.041, *p* < .001; adjusted *R^2^* = .508 with a large effect. The first predictor block (age and BMI) was significant, *ΔF*(2, 91) = 5.757, *p* = .004, *ΔR^2^* = .112 with a small effect. The addition of GWI status on the second block accounted for significant incremental variance, *ΔF*(1, 90) = 35.317, *p* < .001, *ΔR^2^* = .250, medium effect. The addition of PTSD symptom level produced significant incremental variance, *ΔF*(1, 89) = 31.596, *p* < .001, *ΔR^2^* = .167, medium effect. In the final model, health condition (*r_sp_* = -.159; *r_sp_^2^ =* .025, small effect) and PTSD symptoms (*r_sp_* = -.409; *r_sp_^2^ =* .167, medium effect) were negatively associated with physical health. Age (*r_sp_* = -.129; *r_sp_^2^ =* .017) and BMI (*r_sp_* = -.07; *r_sp_^2^ =* .005) did not produce meaningful results.  Regression models were used (*n* = 97) to investigate the model in predicting role limitations due to physical health (i.e., physical role functioning). The final model was statistically significant, *R^2^* = .591, *F*(4, 92) = 33.289, *p* < .001, adjusted *R^2^* = .574 with a large effect. The first block (Age and BMI) was significant, *R^2^* = .071, *F*(2, 94) = 3.602, *p* = .031, adjusted *R^2^* = .051 (small effect). The addition of GWI status demonstrated a significant incremental increase in variance, *ΔF*(1, 93) = 81.937, *p* < .001, *ΔR^2^* = .435; large effect. The addition level of PTSD symptoms also led to a statistically significant incremental variance, *ΔF*(1, 92) = 19.176, *p* < .001, *ΔR^2^* = .085; small effect. Regarding coefficients in the final model, health condition (*r_sp_* = -.344; *r_sp_^2^ =* .118, small effect) and PTSD symptoms (*r_sp_* = -.292; *r_sp_^2^ =* .085, small effect) were negatively associated with physical role functioning. As before, age (*r_sp_* = -.059; *r_sp_^2^ =* .003) and BMI (*r_sp_* = -.08; *r_sp_^2^ =* .006) did not produce meaningful results.  The full model for RAND SF-36 general health (*n* = 98) was significant, *R^2^* = .622, *F*(4, 93) = 38.253, *p* < .001, adjusted *R^2^* = .606 with a large effect. The first predictor block with age and BMI demonstrated statistical significance, *R^2^* = .095, *F*(2, 95) = 4.994, *p* = .009, adjusted *R^2^* = .076 (small effect). The second block (GWI status) demonstrated a significant incremental increase in variance, *ΔF*(1, 94) = 87.302, *p* < .001, *ΔR^2^* = .436; large effect. The third block (PTSD symptoms) also produced significant incremental variance, *ΔF*(1, 93) = 22.417, *p* < .001, *ΔR^2^* = .091; small effect. Coefficients in the final model had mixed results. Health condition (*r_sp_* = -.336; *r_sp_^2^ =* .113, small effect) and PTSD symptoms (*r_sp_* = -.302; *r_sp_^2^ =* .091, small effect) were negatively associated with general health while age (*r_sp_* = -.087; *r_sp_^2^ =* .008) and BMI (*r_sp_* = -.093; *r_sp_^2^ =* .009) did not produce meaningful results.  The full model (*n* = 92) for RAND SF-36 energy/fatigue (i.e., vitality) was significant, *R^2^* = .694, *F*(4, 87) = 49.302, *p* < .001, adjusted *R^2^* = .680 with a large effect. Block 1 (Age and BMI) produced significance, *R^2^* = .122, *F*(2, 89) = 6.167, *p* = .003, adjusted *R^2^* = .102 (small effect). Block 2, with the addition of GWI status, demonstrated statistically significant incremental variance, *ΔF*(1, 88) = 80.00, *p* < .001, *ΔR^2^* = .418; large effect. Block 3, with the addition PTSD symptom scores, also had significant incremental variance, *ΔF*(1, 87) = 43.751, *p* < .001, *ΔR^2^* = .154; medium effect. Health condition (*r_sp_* = -.288; *r_sp_^2^ =* .083, small effect) and PTSD symptoms (*r_sp_* = -.392; *r_sp_^2^ =* .154, medium effect) were negatively associated with vitality. Age (*r_sp_* = -.059; *r_sp_^2^ =* .003) and BMI (*r_sp_* = -.133; *r_sp_^2^ =* .018) did not produce meaningful results.  The final model (*n* = 97) for RAND SF-36 social functioning was significant, *R^2^* = .670, *F*(4, 92) = 46.800, *p* < .001, adjusted *R^2^* = .656 with a large effect. The first block (Age and BMI) was significant, *R^2^* = .125, *F*(2, 94) = 6.732, *p* = .002, adjusted *R^2^* = .107 (small effect). The addition of GWI status demonstrated a significant incremental increase in variance, *ΔF*(1, 93) = 55.606, *p* < .001, *ΔR^2^* = .327; large effect. The addition of PTSD symptoms also led to a statistically significant incremental variance, *ΔF*(1, 92) = 60.837, *p* < .001, *ΔR^2^* = .218; medium effect. Regarding coefficients in the final model, health condition (*r_sp_* = -.167; *r_sp_^2^ =* .028, small effect) and PTSD symptoms (*r_sp_* = -.467; *r_sp_^2^ =* .218, medium effect) were negatively associated with social functioning. Consistent with other subscales, age (*r_sp_* = -.084 *r_sp_^2^ =* .007) and BMI (*r_sp_* = -.12; *r_sp_^2^ =* .014) did not produce meaningful results.  The full model (*n* = 97) for RAND SF-36 role limitations due to emotional problems (i.e., emotional role functioning) was significant, *R^2^* = .666, *F*(4, 92) = 45.808, *p* < .001, adjusted *R^2^* = .651 with a large effect. The first predictor block (age and BMI) was nonsignificant, *ΔF*(2, 94) = 2.933, *p* = .058, *ΔR^2^* = .059 but demonstrated a small effect. The addition of GWI status on the second block accounted for significant incremental variance, *ΔF*(1, 93) = 57.263, *p* < .001, *ΔR^2^* = .359, large effect. The addition of PTSD symptom level produced significant incremental variance, *ΔF*(1, 92) = 68.341, *p* < .001, *ΔR^2^* = .248, medium effect. In the final model, health condition (*r_sp_* = -.169; *r_sp_^2^ =* .029, small effect) and PTSD symptoms (*r_sp_* = -.498; *r_sp_^2^ =* .25, medium effect) were negatively associated with emotional role functioning. Age (*r_sp_* = .009; *r_sp_^2^ =* .0001) and BMI (*r_sp_* = -.061; *r_sp_^2^ =* .004) did not produce meaningful results.  The final model (*n* = 97) for RAND SF-36 emotional well-being (i.e., mental health) was significant, *R^2^* = .679, *F*(4, 92) = 48.576, *p* < .001, adjusted *R^2^* = .665 with a large effect. Block 1 (Age and BMI) produced significance, *R^2^* = .073, *F*(2, 94) = 3.691, *p* = .029, adjusted *R^2^* = .053 (small effect). Block 2, with the addition of GWI status, demonstrated statistically significant incremental variance, *ΔF*(1, 93) = 78.678, *p* < .001, *ΔR^2^* = .425; large effect. Block 3, with the addition PTSD symptom scores, also had significant incremental variance, *ΔF*(1, 92) = 51.803, *p* < .001, *ΔR^2^* = .181; medium effect. Health condition (*r_sp_* = -.246; *r_sp_^2^ =* .061, small effect) and PTSD symptoms (*r_sp_* = -.425; *r_sp_^2^ =* .181, medium effect) were negatively associated with mental health. Age (*r_sp_* = .01; *r_sp_^2^ =* .0001) and BMI (*r_sp_* = -.09; *r_sp_^2^ =* .0081) did not produce meaningful results.  The final model (*n* = 97) for RAND SF-36 pain was significant, *R^2^* = .612, *F*(4, 92) = 36.295, *p* < .001, adjusted *R^2^* = .595 with a large effect. The first predictor block with age and BMI demonstrated statistical significance, *R^2^* = .111, *F*(2, 94) = 5.856, *p* = .004, adjusted *R^2^* = .092 (small effect). The second block (GWI status) demonstrated a significant incremental increase in variance, *ΔF*(1, 93) = 56.954, *p* < .001, *ΔR^2^* = .431; large effect. The third block (PTSD symptoms) also produced significant incremental variance, *ΔF*(1, 92) = 38.798, *p* < .001, *ΔR^2^* = .164; medium effect. Coefficients in the final model had unique results. Health condition (*r_sp_* = -.213; *r_sp_^2^ =* .045, small effect), PTSD symptoms (*r_sp_* = -.404; *r_sp_^2^ =* .163, medium effect), and BMI (*r_sp_* = -.154; *r_sp_^2^ =* .024) were negatively associated with bodily pain while age (*r_sp_* = -.032; *r_sp_^2^ =* .001) did not produce meaningful results.  **MFI Results:** The second set of hierarchical regressions was run with available data (*n* = 97) to investigate if health status (GWI versus healthy control) and PTSD symptom level improved the prediction of MFI scores over and above age and BMI. Fatigue, as measured by the MFI, was divided between five subscales: General Fatigue, Physical Fatigue, Mental Fatigue, Reduced Motivation, and Reduced Activity. All the subscales were analyzed in separate models. Predictor blocks were held constant across models: Block 1 (Age and BMI), Block 2 (GWI status), and Block 3 (PTSD symptom level).  The full model (*n* = 96) for MFI physical fatigue was significant, *R^2^* = .590, *F*(4, 91) = 32.676, *p* < .001; adjusted *R^2^* = .571 with a large effect. The first predictor block (age and BMI) was significant, *ΔF*(2, 93) = 5.493, *p* = .006, *ΔR^2^* = .106 with a small effect. The addition of GWI status on the second block accounted for significant incremental variance, *ΔF*(1, 92) = 80.731, *p* < 0.001, *ΔR^2^* = .418, large effect. The addition of PTSD symptom level produced significant incremental variance, *ΔF*(1, 91) = 14.608, *p* < .001, *ΔR^2^* = .066, small effect. In the final model, health condition (*r_sp_* = .35; *r_sp_^2^ =* .123, small effect) and PTSD symptoms (*r_sp_* = .257; *r_sp_^2^ =* .066, small effect) were positively associated with physical fatigue. Age (*r_sp_* = .075; *r_sp_^2^ =* .006) and BMI (*r_sp_* = .257; *r_sp_^2^ =* .066) did not produce meaningful results.  The full model for MFI Mental Fatigue (*n* = 97) was significant, *R^2^* = .651, *F*(4, 92) = 42.989, *p* < .001, adjusted *R^2^* = .636 with a large effect. The first predictor block with age and BMI demonstrated statistical significance, *R^2^* = .081, *F*(2, 94) = 4.127, *p* = .019, adjusted *R^2^* = .061 (small effect). The second block (GWI status) demonstrated a significant incremental increase in variance, *ΔF*(1, 93) = 84.845, *p* < .001, *ΔR^2^* = .439; large effect. The third block (PTSD symptoms) also produced significant incremental variance, *ΔF*(1, 92) = 34.888, *p* < .001, *ΔR^2^* = .132; medium effect. Health condition (*r_sp_* = .304; *r_sp_^2^ =* .092, small effect) and PTSD symptoms (*r_sp_* = .364; *r_sp_^2^ =* .132, medium effect) were positively associated with mental fatigue while age (*r_sp_* = .095; *r_sp_^2^ =* .009) and BMI (*r_sp_* = .035; *r_sp_^2^ =* .001) did not produce meaningful results.  The full model (*n* = 94) for MFI Reduced Activity was significant, *R^2^* = .564, *F*(4, 89) = 28.814, *p* < .001, adjusted *R^2^* = .545 with a large effect. Block 1 (Age and BMI) produced significance, *R^2^* = .155, *F*(2, 91) = 8.329, *p* = < .001, adjusted *R^2^* = .136 (medium effect). Block 2, with the addition of GWI status, demonstrated statistically significant incremental variance, *ΔF*(1, 90) = 64.943, *p* < .001, *ΔR^2^* = .354; large effect. Block 3, with the addition PTSD symptom scores, also had significant incremental variance, *ΔF*(1, 89) = 11.287, *p* < .001, *ΔR^2^* = .055; small effect. Age (*r_sp_* = .159; *r_sp_^2^ =* .025, small effect), health condition (*r_sp_* = .33; *r_sp_^2^ =* .109, small effect) and PTSD symptoms (*r_sp_* = .235; *r_sp_^2^ =* .055, small effect) were positively associated with Reduced Activity. However, BMI (*r_sp_* = .114; *r_sp_^2^ =* .013) did not produce meaningful results.  The final model (*n* = 96) for MFI Reduced Motivation was significant, *R^2^* = .535, *F*(4, 91) = 26.187, *p* < .001, adjusted *R^2^* = .515 with a large effect. The first block (Age and BMI) was significant, *R^2^* = .062, *F*(2, 93) = 3.052, *p* = .052, adjusted *R^2^* = .041 (small effect). The addition of GWI status demonstrated a significant incremental increase in variance, *ΔF*(1, 92) = 52.999, *p* < .001, *ΔR^2^* = .343; large effect. The addition level of PTSD symptoms also led to a statistically significant incremental variance, *ΔF*(1, 91) = 25.549, *p* < .001, *ΔR^2^* = .131; medium effect. Regarding coefficients in the final model, health condition (*r_sp_* = .24; *r_sp_^2^ =* .058, small effect) and PTSD symptoms (*r_sp_* = .361; *r_sp_^2^ =* .130, medium effect) were positively associated with Reduced Motivation. Age (*r_sp_* = .06l; *r_sp_^2^ =* .004) and BMI (*r_sp_* = .03; *r_sp_^2^ =* .001) did not produce meaningful results.  The full model (*n* = 96) for MFI General Fatigue was significant, *R^2^* = .690, *F*(4, 91) = 50.728, *p* < .001, adjusted *R^2^* = .677 with a large effect. The first predictor block (Age and BMI) was significant, *ΔF*(2, 93) = 5.818, *p* = .004, *ΔR^2^* = .111 with a small effect. The addition of GWI status on the second block accounted for significant incremental variance, *ΔF*(1, 92) = 93.630, *p* < .001, *ΔR^2^* = .448, large effect. The addition of PTSD symptom level produced significant incremental variance, *ΔF*(1, 91) = 38.469, *p* < .001, *ΔR^2^* = .131, medium effect. In the final model, health condition (*r_sp_* = .306; *r_sp_^2^ =* .094, small effect) and PTSD symptoms (*r_sp_* = .362; *r_sp_^2^ =* .131, medium effect) were positively associated with General Fatigue. Age (*r_sp_* = .03; *r_sp_^2^ =* .0001) and BMI (*r_sp_* = .139; *r_sp_^2^ =* .019) did not produce meaningful results.  **Table S3: RAND SF-36 Physical Functioning** | | | | | | |
| --- | --- | --- | --- | --- | --- | --- |
|  | Model 1 | | Model 2 | | Model 3 | |
| Variable | B | β | B | β | B | β |
| Constant | 155.341 |  | 156.129 |  | 135.583 |  |
| Age | -1.197 | -.245 | -1.264 | -.259 | -.677 | -.139 |
| BMI | -1.187 | -.177 | -.632 | -.094 | -.493 | -.073 |
| Health Status ^a^ |  |  | -30.404 | -.507 | -12.042 | -.201 |
| Trauma Level ^b^ |  |  |  |  | -.360 | -.529 |
|  |  |  |  |  |  |  |
| *R^2^* | .112* |  | .362** |  | .530** |  |
| *R^2 adj^* | .093* |  | .341** |  | .508** |  |
| *F* | 5.757* |  | 17.057** |  | 25.041** |  |
| *Δ R^2^* | .112* |  | .250** |  | .167** |  |
| *Δ F* | 5.757* |  | 35.317** |  | 31.596** |  |

*Note. *p < .05, **p <.001,*^a^ Case designation (GWI or HC), ^b^Trauma level (PTSD-related symptoms from DTS total), B indicates unstandardized coefficients, β indicates standardized coefficients

| **Table S4: RAND SF-36 Role Limitations due to Physical Health** | | | | | | |
| --- | --- | --- | --- | --- | --- | --- |
|  | Model 1 | | Model 2 | | Model 3 | |
| Variable | B | β | B | β | B | β |
| Constant | 154.610 |  | 157.365 |  | 135.613 |  |
| Age | -.928 | -.129 | -1.060 | -.148 | -.455 | -.064 |
| BMI | -2.001 | -.205 | -.965 | -.099 | -.814 | -.084 |
| Health Status ^a^ |  |  | -59.046 | -.668 | -38.871 | -.440 |
| Trauma Level ^b^ |  |  |  |  | -.378 | -.383 |
|  |  |  |  |  |  |  |
| *R^2^* | .071 |  | .506** |  | .591** |  |
| *R^2 adj^* | .051 |  | .490** |  | .574** |  |
| *F* | 3.602 |  | 31.782** |  | 33.289** |  |
| *Δ R^2^* | .071 |  | .435** |  | .085** |  |
| *Δ F* | 3.602 |  | 81.937** |  | 19.176** |  |

*Note. *p < .05, **p <.001,*^a^ Case designation (GWI or HC), ^b^Trauma level (PTSD-related symptoms from DTS total), B indicates unstandardized coefficients, β indicates standardized coefficients

| **Table S5: RAND SF-36 General Health Perceptions** | | | | | | |
| --- | --- | --- | --- | --- | --- | --- |
|  | Model 1 | | Model 2 | | Model 3 | |
| Variable | B | β | B | β | B | β |
| Constant | 130.582 |  | 131.174 |  | 116.368 |  |
| Age | -.753 | -.161 | -.839 | -.180 | -.432 | -.092 |
| BMI | -1.437 | -.228 | -.718 | -.114 | -.611 | -.097 |
| Health Status ^a^ |  |  | -38.362 | -.669 | -24.731 | -.432 |
| Trauma Level ^b^ |  |  |  |  | -.255 | -.398 |
|  |  |  |  |  |  |  |
| *R^2^* | .095** |  | .531** |  | .622** |  |
| *R^2 adj^* | .076** |  | .516** |  | .606** |  |
| *F* | 4.994** |  | 35.454** |  | 38.253** |  |
| *Δ R^2^* | .095** |  | .436** |  | .091** |  |
| *Δ F* | 4.994** |  | 87.302** |  | 22.417** |  |

*Note. *p < .05, **p <.001,*^a^ Case designation (GWI or HC), ^b^Trauma level (PTSD-related symptoms from DTS total), B indicates unstandardized coefficients, β indicates standardized coefficients

| **Table S6: RAND SF-36 Energy/Fatigue** | | | | | | |
| --- | --- | --- | --- | --- | --- | --- |
|  | Model 1 | | Model 2 | | Model 3 | |
| Variable | B | β | B | β | B | β |
| Constant | 124.417 |  | 123.674 |  | 110.148 |  |
| Age | -.437 | -.092 | -.659 | -.139 | -.292 | -.062 |
| BMI | -1.959 | -.318 | -1.030 | -.167 | -.860 | -.140 |
| Health Status ^a^ |  |  | -36.599 | -.664 | -20.230 | -.367 |
| Trauma Level ^b^ |  |  |  |  | -.317 | -.502 |
|  |  |  |  |  |  |  |
| *R^2^* | .122** |  | .540** |  | .694** |  |
| *R^2 adj^* | .102** |  | .524** |  | .680** |  |
| *F* | 6.167** |  | 34.427** |  | 49.302** |  |
| *Δ R^2^* | .122** |  | .418** |  | .154** |  |
| *Δ F* | 6.167** |  | 80** |  | 43.751** |  |

*Note. *p < .05, **p <.001,*^a^ Case designation (GWI or HC), ^b^Trauma level (PTSD-related symptoms from DTS total), B indicates unstandardized coefficients, β indicates standardized coefficients

| **Table S7: RAND SF-36 Social Functioning** | | | | | | |
| --- | --- | --- | --- | --- | --- | --- |
|  | Model 1 | | Model 2 | | Model 3 | |
| Variable | B | β | B | β | B | β |
| Constant | 166.029 |  | 167.838 |  | 141.493 |  |
| Age | -1.134 | -.209 | -1.220 | -.225 | -.488 | -.090 |
| BMI | -1.783 | -.242 | -1.103 | -.149 | -.920 | -.125 |
| Health Status ^a^ |  |  | -38.782 | -.579 | -14.345 | -.214 |
| Trauma Level ^b^ |  |  |  |  | -.458 | -.612 |
|  |  |  |  |  |  |  |
| *R^2^* | .125** |  | .453** |  | .670** |  |
| *R^2 adj^* | .107** |  | .435** |  | .656** |  |
| *F* | 6.732** |  | 25.630* |  | 46.800** |  |
| *Δ R^2^* | .125** |  | .327** |  | .218** |  |
| *Δ F* | 6.732** |  | 55.606** |  | 60.837** |  |

*Note. *p < .05, **p <.001,*^a^ Case designation (GWI or HC), ^b^Trauma level (PTSD-related symptoms from DTS total), B indicates unstandardized coefficients, β indicates standardized coefficients

| **Table S8: RAND SF-36 Role limitations due to emotional problems** | | | | | | |
| --- | --- | --- | --- | --- | --- | --- |
|  | Model 1 | | Model 2 | | Model 3 | |
| Variable | B | β | B | β | B | β |
| Constant | 151.542 |  | 154.012 |  | 117.340 |  |
| Age | -.835 | -.118 | -.952 | -.134 | .067 | .009 |
| BMI | -.1.793 | -.186 | -.864 | -.090 | -.609 | -.063 |
| Health Status ^a^ |  |  | -52.940 | -.606 | -18.925 | -.217 |
| Trauma Level ^b^ |  |  |  |  | -.637 | -.654 |
|  |  |  |  |  |  |  |
| *R^2^* | .059 |  | .417** |  | .666** |  |
| *R^2 adj^* | .039 |  | .399** |  | .651** |  |
| *F* | 2.933 |  | 22.213** |  | 45.808** |  |
| *Δ R^2^* | .059 |  | .359** |  | .248** |  |
| *Δ F* | 2.933 |  | 57.263** |  | 68.341** |  |

*Note. *p < .05, **p <.001,*^a^ Case designation (GWI or HC), ^b^Trauma level (PTSD-related symptoms from DTS total), B indicates unstandardized coefficients, β indicates standardized coefficients

| **Table S9: RAND SF-36 Emotional Well-Being** | | | | | | |
| --- | --- | --- | --- | --- | --- | --- |
|  | Model 1 | | Model 2 | | Model 3 | |
| Variable | B | β | B | β | B | β |
| Constant | 119.011 |  | 117.549 |  | 101.191 |  |
| Age | -.382 | -.096 | -.431 | -.109 | .043 | .011 |
| BMI | -1.235 | -.231 | -.605 | -.113 | -.502 | -.094 |
| Health Status ^a^ |  |  | -32.267 | -.662 | -15.536 | -.319 |
| Trauma Level ^b^ |  |  |  |  | -.306 | -.564 |
|  |  |  |  |  |  |  |
| *R^2^* | .073* |  | .498** |  | .679** |  |
| *R^2 adj^* | .053* |  | .482** |  | .665** |  |
| *F* | 3.691* |  | 30.720** |  | 48.576** |  |
| *Δ R^2^* | .073* |  | .425** |  | .181** |  |
| *Δ F* | 3.691* |  | 78.678** |  | 51.803** |  |

*Note. *p < .05, **p <.001,*^a^ Case designation (GWI or HC), ^b^Trauma level (PTSD-related symptoms from DTS total), B indicates unstandardized coefficients, β indicates standardized coefficients

| **Table S10: RAND SF-36 Pain** | | | | | | |
| --- | --- | --- | --- | --- | --- | --- |
|  | Model 1 | | Model 2 | | Model 3 | |
| Variable | B | β | B | β | B | β |
| Constant | 141.160 |  | 142.849 |  | 121.870 |  |
| Age | -.671 | -.134 | -.751 | -.151 | -.168 | -.034 |
| BMI | -1.866 | -.275 | -1.231 | -.181 | -1.085 | -.160 |
| Health Status ^a^ |  |  | -36.205 | -.588 | -16.746 | -.272 |
| Trauma Level ^b^ |  |  |  |  | -.365 | -.531 |
|  |  |  |  |  |  |  |
| *R^2^* | .111** |  | .449** |  | .612** |  |
| *R^2 adj^* | .092** |  | .431** |  | .595** |  |
| *F* | 5.856** |  | 25.213** |  | 36.295** |  |
| *Δ R^2^* | .111** |  | .338** |  | .164** |  |
| *Δ F* | 5.856** |  | 56.954** |  | 38.789** |  |

*Note. *p < .05, **p <.001,*^a^ Case designation (GWI or HC), ^b^Trauma level (PTSD-related symptoms from DTS total), B indicates unstandardized coefficients, β indicates standardized coefficients

| **Table S11: MFI Physical Fatigue** | | | | | | |
| --- | --- | --- | --- | --- | --- | --- |
|  | Model 1 | | Model 2 | | Model 3 | |
| Variable | B | β | B | β | B | β |
| Constant | -43.209 |  | -41.065 |  | -28.186 |  |
| Age | .757 | .151 | .766 | .153 | .400 | .080 |
| BMI | 1.696 | .254 | .968 | .145 | .878 | .132 |
| Health Status ^a^ |  |  | 40.162 | .656 | 27.622 | .451 |
| Trauma Level ^b^ |  |  |  |  | .233 | .340 |
|  |  |  |  |  |  |  |
| *R^2^* | .106** |  | .524** |  | .590** |  |
| *R^2 adj^* | .086** |  | .508** |  | .571** |  |
| *F* | 5.493** |  | 33.712** |  | 32.676** |  |
| *Δ R^2^* | .106** |  | .418** |  | .066** |  |
| *Δ F* | 5.493** |  | 80.731** |  | 14.608** |  |

*Note. *p < .05, **p <.001,*^a^ Case designation (GWI or HC), ^b^Trauma level (PTSD-related symptoms from DTS total), B indicates unstandardized coefficients, β indicates standardized coefficients

| **Table S12: MFI Mental Fatigue** | | | | | | |
| --- | --- | --- | --- | --- | --- | --- |
|  | Model 1 | | Model 2 | | Model 3 | |
| Variable | B | β | B | β | B | β |
| Constant | -38.494 |  | -39.247 |  | -18.477 |  |
| Age | 1.030 | .190 | 1.126 | .207 | .553 | .102 |
| BMI | 1.261 | .172 | .418 | .057 | .265 | .036 |
| Health Status ^a^ |  |  | 44.982 | .672 | 26.017 | .389 |
| Trauma Level ^b^ |  |  |  |  | .358 | .477 |
|  |  |  |  |  |  |  |
| *R^2^* | .081* |  | .519** |  | .651** |  |
| *R^2 adj^* | .061* |  | .504** |  | .636** |  |
| *F* | 4.127* |  | 33.487** |  | 42.989* |  |
| *Δ R^2^* | .081* |  | .439** |  | .132** |  |
| *Δ F* | 4.127* |  | 84.845** |  | 34.888** |  |

*Note. *p < .05, **p <.001,*^a^ Case designation (GWI or HC), ^b^Trauma level (PTSD-related symptoms from DTS total), B indicates unstandardized coefficients, β indicates standardized coefficients

| **Table S13: MFI Reduced Activity** | | | | | | |
| --- | --- | --- | --- | --- | --- | --- |
|  | Model 1 | | Model 2 | | Model 3 | |
| Variable | B | β | B | β | B | β |
| Constant | -64.434 |  | -57.128 |  | -43.373 |  |
| Age | 1.326 | .268 | 1.182 | .239 | .840 | .170 |
| BMI | 1.470 | .232 | .874 | .138 | .753 | .119 |
| Health Status ^a^ |  |  | 35.025 | .604 | 24.427 | .422 |
| Trauma Level ^b^ |  |  |  |  | .200 | .313 |
|  |  |  |  |  |  |  |
| *R^2^* | .155** |  | .509** |  | .564** |  |
| *R^2 adj^* | .136** |  | .493** |  | .545** |  |
| *F* | 8.329** |  | 31.102** |  | 28.814** |  |
| *Δ R^2^* | .155** |  | .354** |  | .055** |  |
| *Δ F* | 8.329** |  | 64.943** |  | 11.287** |  |

*Note. *p < .05, **p <.001,*^a^ Case designation (GWI or HC), ^b^Trauma level (PTSD-related symptoms from DTS total), B indicates unstandardized coefficients, β indicates standardized coefficients

| **Table S14: MFI Reduced Motivation** | | | | | | |
| --- | --- | --- | --- | --- | --- | --- |
|  | Model 1 | | Model 2 | | Model 3 | |
| Variable | B | β | B | β | B | β |
| Constant | -23.080 |  | -22.517 |  | -4.547 |  |
| Age | .737 | .165 | .770 | .173 | .287 | .064 |
| BMI | .900 | .151 | .310 | .052 | .185 | .031 |
| Health Status ^a^ |  |  | 32.623 | .594 | 16.935 | .308 |
| Trauma Level ^b^ |  |  |  |  | .294 | .478 |
|  |  |  |  |  |  |  |
| *R^2^* | .062* |  | .405** |  | .535** |  |
| *R^2 adj^* | .041* |  | .385** |  | .515** |  |
| *F* | 3.052* |  | 20.839** |  | 26.187** |  |
| *Δ R^2^* | .062* |  | .343** |  | .131** |  |
| *Δ F* | 3.052* |  | 52.999** |  | 25.549** |  |

*Note. *p < .05, **p <.001,*^a^ Case designation (GWI or HC), ^b^Trauma level (PTSD-related symptoms from DTS total), B indicates unstandardized coefficients, β indicates standardized coefficients

| **Table S15: MFI General Fatigue** | | | | | | |
| --- | --- | --- | --- | --- | --- | --- |
|  | Model 1 | | Model 2 | | Model 3 | |
| Variable | B | β | B | β | B | β |
| Constant | -40.036 |  | -32.777 |  | -17.767 |  |
| Age | .618 | .123 | .620 | .123 | .157 | .031 |
| BMI | 1.961 | .289 | 1.052 | .155 | .977 | .144 |
| Health Status ^a^ |  |  | 41.153 | .683 | 23.832 | .395 |
| Trauma Level ^b^ |  |  |  |  | .321 | .475 |
|  |  |  |  |  |  |  |
| *R^2^* | .111** |  | .560** |  | .690** |  |
| *R^2 adj^* | .092** |  | .545** |  | .677** |  |
| *F* | 5.818** |  | 38.951** |  | 50.728** |  |
| *Δ R^2^* | .111** |  | .448** |  | .131** |  |
| *Δ F* | 5.818** |  | 93.630** |  | 38.469** |  |

*Note. *p < .05, **p <.001,*^a^ Case designation (GWI or HC), ^b^Trauma level (PTSD-related symptoms from DTS total), B indicates unstandardized coefficients, β indicates standardized coefficients
